# Protein and lipid homeostasis altered in rat macrophages after exposure to metallic oxide nanoparticles

**DOI:** 10.1007/s10565-019-09484-6

**Published:** 2019-07-27

**Authors:** Zahra Doumandji, Ramia Safar, Mélanie Lovera-Leroux, Sara Nahle, Hilary Cassidy, David Matallanas, Bertrand Rihn, Luc Ferrari, Olivier Joubert

**Affiliations:** 1 Institut Jean Lamour, UMR 7198, CNRS-Université de Lorraine, 2 allée André Guinier, BP 50840, 54011 Nancy, France; 2 Faculté de Médecine, INSERM UMR_S NGERE 954, Université de Lorraine, Vandœuvre-lès-Nancy, France; 3 Conway Institute of Biomolecular and Biomedical Research, University College Dublin, Dublin, Ireland

**Keywords:** Zinc oxide nanoparticles, Zinc iron oxide nanoparticles, Iron oxide nanoparticles, Transcriptomics, Proteomics, NR8383

## Abstract

Metal oxide nanoparticles (NPs), such as ZnO, ZnFe_2_O_4_, and Fe_2_O_3_, are widely used in industry. However, little is known about the cellular pathways involved in their potential toxicity. Here, we particularly investigated the key molecular pathways that are switched on after exposure to sub-toxic doses of ZnO, ZnFe_2_O_4_, and Fe_2_O_3_ in the in vitro rat alveolar macrophages (NR8383). As in our model, the calculated IC_50_ were respectively 16, 68, and more than 200 μg/mL for ZnO, ZnFe_2_O_4_, and Fe_2_O_3_; global gene and protein expression profiles were only analyzed after exposure to ZnO and ZnFe_2_O_4_ NPs. Using a rat genome microarray technology, we found that 985 and 1209 genes were significantly differentially expressed in NR8383 upon 4 h exposure to ¼ IC_50_ of ZnO and ZnFe_2_O_4_ NPs, respectively. It is noteworthy that metallothioneins were overexpressed genes following exposure to both NPs. Moreover, Ingenuity Pathway Analysis revealed that the top canonical pathway disturbed in NR8383 exposed to ZnO and ZnFe_2_O_4_ NPs was eIF2 signaling involved in protein homeostasis. Quantitative mass spectrometry approach performed from both NR8383 cell extracts and culture supernatant indicated that 348 and 795 proteins were differentially expressed upon 24 h exposure to ¼ IC_50_ of ZnO and ZnFe_2_O_4_ NPs, respectively. Bioinformatics analysis revealed that the top canonical pathways disturbed in NR8383 were involved in protein homeostasis and cholesterol biosynthesis for both exposure conditions. While VEGF signaling was specific to ZnO exposure, iron homeostasis signaling pathway was specific to ZnFe_2_O_4_ NPs. Overall, the study provides resource of transcriptional and proteomic markers of response to ZnO and ZnFe_2_O_4_ NP-induced toxicity through combined transcriptomics, proteomics, and bioinformatics approaches.

**Electronic supplementary material:**

The online version of this article (10.1007/s10565-019-09484-6) contains supplementary material, which is available to authorized users.

## Introduction

At the nanoscale, matter is governed by quantum mechanics and could have new chemical and physical properties, different from the molecular counterpart (Schins et al. 2004). When the tiny size allows the nanoparticles (NPs) to be introduced into biological organisms by cellular internalization, the interaction NP-living takes place. This ability to interact makes NP a potent means of delivering and transporting substances at the cellular level to treat on a very small scale. In this way, industries tend to optimize new biological and biochemical applications. Contrariwise, the large specific surface area of NPs could induce severe adverse effects on the living, compared with their homologous macroscopic materials. Therefore, a deep analysis of cellular responses to nanomaterials is necessary before they can be safely used. It is in this perspective that we have studied the cytotoxic potential of ZnO, ZnFe_2_O_4_, and Fe_2_O_3_ NPs.

Nanoparticles of metal oxides are expressed, used, and consumed in large quantities in many countries. They are part of components of cosmetics, medical, electronics, and food products. Consequently, humans are repeatedly exposed to various NPs via inhalation (respiratory), ingestion (oral), or dermal (cutaneous) routes.

Their wide use must be challenged for the potential negative effects they can produce. Thus, it is urgent to assess the risk of different exposures to nanoparticles. Indeed, the nanoparticle-living interaction could result in biological damages (Alhadlaq et al. 2015; Eidi et al. 2012; Singh and Lillard 2009; Yang et al. 2015).

Industry’s reliance on nanotechnology involves the inhalation exposure of workers to multiple NPs within their manufactures. Although the human body has several barriers against the penetration of foreign inhaled substances, such as the nose that acts as a filter, some nanoparticles could be trapped in the pulmonary alveolar region (Oberdörster et al. 2005; Présumé et al. 2016). Thus, an interaction between NPs and pulmonary alveolar cells could occur (Buzea et al. 2007). In this context, this study allows us to analyze the cellular and molecular responses linked to the exposure of rat pulmonary alveolar cells (NR8383) to both ZnO and ZnFe_2_O_4_ NPs. NR8383 cells are relevant due to their immune functions and their ability of contact with foreign bodies (Hussain et al. 2012). In addition, experimental rat or mouse models have been validated by many studies as toxicity paradigm of airways (Ganguly et al. 2017; Gaté et al. 2017; Wallin et al. 2017). Moreover, in order to respect the three “R” of the ethical approach described by Russel and Burch in 1959 for reducing, replacing, and refining the use of animal testing, it seems important to develop in vitro models (Tannenbaum and Bennett 2015). In vitro study of different cell lines represents a promising tool for the implementing predictive devices of NPs (Alhadlaq et al. 2015). The present cellular model will help establish a correlation of deregulated genes and their associated molecular pathways and protein production to reveal the accentuated response biomarkers of ZnO and ZnFe_2_O_4_ NPs on that particular biological system.

The potential toxic effects of ZnO NP are known to be related to their solubility (Prach et al. 2013). Indeed, Zn ions could have negative impact on cellular homeostasis because there is a large panel of metalloproteins which are zinc dependent (Andreini et al. 2006), e.g., transcription factors. Studies have shown that Fe_2_O_3_ NPs can induce cell death (Brunner et al. 2006), while other studies showed that Fe_2_O_3_ induces no cytotoxicity (Chusuei et al. 2013). The ZnFe_2_O_4_ NPs were the least studied of the NP panel studied here. Thus, there is a lack in the literature on the potential toxic effects of ZnFe_2_O_4_. In this paper, this study provides information about the cytotoxic potential and for the first time the gene expression profile and proteome changes of cells exposed to ZnFe_2_O_4_ NPs.

The aim of this study is (i) to evaluate and compare the cytotoxicity of ZnO, ZnFe_2_O_4_, and Fe_2_O_3_ NPs in rat macrophage alveolar NR8383 cells, (ii) to measure the deliverable dose of NPs to NR8383 cells, and (iii) to analyze in these cells the consequence of sub-toxic NPs exposure on transcriptome and proteome profiles.

## Materials and methods

### Nanoparticles

Uncoated zinc oxide (ZnO) NPs were obtained from the Joint Research Center (JRCNM110). ZnO NPs main physicochemical characteristics were as follows: primary particle size of 158 nm; specific surface area of 12 m^2^/g (Table 1). The zinc ferrite oxide NPs (NanoAmor® ZnFe_2_O_4_, NRCWE-021) were obtained from Dr. Ulla Vogel (National Research Center for the Working Environment, NRCWE, Copenhagen). The main physicochemical characteristics of NanoAmor® ZnFe_2_O_4_ NP containing 5–10 wt.% ZnO and 10–15 wt.% Fe_2_O_3_ were as follows: particle size of 15–30 nm and specific surface area of 87.7 m^2^/g (Table 1). The iron oxide NPs (NanoAmor Fe_2_O_3_, NRCWE-018) were also from NRCWE (Copenhagen) with the following main physicochemical properties: particle size of 30–50 nm; specific surface area of 27.7 m^2^/g (Table 1).

**Table 1:** Physical-chemical properties of nanoparticles studied by Joint Research Center (JRC) or the National Research Centre for the Working Environment (NRCWE), in Denmark, Copenhagen

	ZnO	ZnFe_2_O_3_	Fe_2_O_3_
NM-Code	NM-110	NRCWE-021	NRCWE-018
Manufacturer	JRC	NanoAmor	NanoAmor
Cat. Num.	JRCNM01100a	5710FY	2520ZH
Surface area (m^2^/g)	12	87.7	27.7
Purity (%)	Low impurities	98.50%	99%
Crystal form	Zincite	Cubic	Alpha

### Characterization of ZnO, ZnFe_2_O_4_, and Fe_2_O_3_ nanoparticles

Dry nanopowders are extemporaneously suspended in deionized water at 2.56 mg/mL and then directly sonicated (0.4 mm, *Hielsher Ultrasonics*) at 4 °C for 6 min at 30% of amplitude or 161 J/mL (Cohen et al. 2013). Then, NPs were diluted to the desired concentrations in cell media (DMEM, Sigma-D1145) without fetal bovine serum (FBS). The average hydrodynamic size and the zeta potential of each NP were determined by dynamic light scattering (DLS) on a ZetaSizer™ (Malvern instruments, Malvern, UK). The characterizations on DLS were done directly after suspension in cell media at 6.25 μg/mL, 50 μg/mL, and 200 μg/mL.

The shape of the NPs was characterized by transmission electron microscopy (TEM). A drop of aqueous suspension of each NPs was poured onto carbo-coated copper grid and air-dried for transmission electron microscopy observations (ARM 200F microscope operating at 200 kV).

### Cell culture and exposure

NR8383 alveolar rat macrophage cell line was obtained from the American Type Culture Collection (ATCC, USA) and was grown in DMEM supplemented with 15% heat-inactivated FBS, 4 mM L-glutamine (SIGMA-G7513), and a mixture of antibiotic/antimycotic composed of 100 U/mL of penicillin, 100 μg/mL of streptomycin (SIGMA-P0781), and 0.25 μg/mL of amphotericin B (SIGMA-A2942), at 37 °C in a humidified mixture of air (95%) and CO_2_ (5%).

For all experiments, cells were seeded 24 h before exposure to NPs at a density of 5 × 10^4^ cells/mL. Cells were exposed to NPs in cell media without FBS. The different concentrations of NPs were mixed at room temperature to ensure homogeneity of the samples before exposure to cells. Cells not exposed to NPs served as controls in each experiment.

### Cell viability

*Lactate dehydrogenase (LDH) leakage* was analyzed using the LDH assay (Roche-4744934001, Germany) following the manufacturer’s instruction. Briefly, NR8383 cells were seeded at 5 × 10^4^ cells/mL in 96-well plates and exposed to different concentrations ranging 0.2 and 7.2 cm^2^ of ZnO NPs per square centimeters of cells (cm^2^/cm^2^), 1.7 and 53.1 cm^2^/cm^2^ of ZnFe_2_O_4_, and 0.5 and 16.8 cm^2^/cm^2^ of Fe_2_O_3_ NPs. These specific surface concentrations are equivalent of mass concentrations of 6.25 and 200 μg/mL. After 24 h of exposure, plates were centrifuged at 800×*g* for 10 min and 100 μL of each supernatant were transferred to a new 96-well plate with a black bottom that was already prefilled with 100 μL of the LDH reaction mixture. Extracellular medium was incubated for 30 min at room temperature, then 50 μL of a stop solution was added and the absorbance was measured at 490 nm on a microplate reader. NR8383 cells treated with 5% Triton were considered as positive control. Unexposed NR8383 cells were considered as negative control. NP cytotoxicity was expressed as the percent of LDH leakage measured in positive control cells. Dose-effect relationships were assessed by ANOVA and Dunett’s test. *p* values < 0.05 were considered significant.

*Metabolic activity* was assessed using the WST-1 assay (Berridge et al. 1996) (Roche, 11644807001, USA), according to manufacturer’s protocol. NR8383 cells were seeded at 5 × 10^4^ cells/mL in 96-well plates and exposed to different concentrations (6.25 to 200 μg/mL) of ZnO, ZnFe_2_O_4_, and Fe_2_O_3_ NPs. After 24 h of exposure, WST-1 reagent was added to each well. Cells were incubated at 37 °C for 2 h. The absorbance of the solution was determined at 480 nm on a microreader (BioRad-iMARK). IC_50_ was measured for each type of NPs according to Reed-Muench method (Reed and Muench 1938) from WST-1 results.

### Measure of internal dosimetry by ICP-OES

Experiments were conducted on NR8383 cells to determine the amount of cytotoxic NPs in contact with the cell layers, whether inside or on the cells, after 24 h of exposure. In order to obtain the amount of material for reliable elementary determinations, the experiment was carried out on 1 × 10^6^ cells/5 mL in 6-well plates and exposed to both ¼ IC_50_ and IC_50_ of ZnO and ZnFe_2_O_4_, knowing that the IC_50_ of ZnO and ZnFe_2_O_4_ on NR8383 were 0.51 (16 μg/mL) and 18.5 cm^2^/cm^2^ (68 μg/mL), respectively. After 24 h of exposure, cells were centrifuged at a low speed of 300×*g* for 10 min, washed with NaCl 0.9%, and resuspended in 500 μL NaCl 0.9%. After mineralization with 12 N HCl in microwaves from room temperature up to 180 °C, and then held at 180 °C for 15 min, Zn and Fe were directly measured by using the 720-ES Inductively Coupled Plasma Optical Emission Spectrometer (ICP-OES) (Varian, Belgium). Mass equivalents of ZnO (MW 81.38) and ZnFe_2_O_4_ (MW 241.07) in contact with cells have been calculated from the amount of Zn (MW 65.38) and Fe (MW 55.8) measured.

### RNA isolation and quantification

To evaluate gene expression, total RNA was isolated from NR8383 cells exposed for 4 h to ¼ IC_50_ of each NP by using the RNA-Solv reagent (R6830-02, USA). Unexposed cells were used as control. RNA content was determined by measuring absorption at 260 nm using a spectrophotometer (Biotech-Biospec-Nano, Shimadzu). Optimal purity of RNA was ensured by determination of the 260/280 nm of an absorbance ratio A260/A280 > 1.8. RNA integrity was confirmed with the Agilent bioanalyzer 2100 and RNA 6000 Pico Labchip kit (Agilent Biotechnologies, Palo Alto, CA). The RNA integrity number (RIN) score cutoff of 8 was used to determine whether the RNA integrity was qualified or not.

### Microarray expression profiling

The microarray experiments were designed to perform four biological replicates for ¼ IC_50_ dose for each cytotoxic NP. The cRNA synthesis from cDNA and Cy3-dye labelling, hybridization, and washing steps were carried out with 100 ng of total RNA following the manufacturer’s specifications (One-Color Microarray-Based Gene Expression Analysis, version 6.6, Agilent Technologies Inc., USA). Microarray slides were scanned by Agilent DNA microarray scanner (G2505C) by setting the following: (i) one color scan channel for 8 × 60 k array slides, (ii) scan area of 61 × 21.6 mm, (iii) scan resolution of 3 μm, (iv) dye channel to Green, (v) Tiff file dynamic range of 20 bits, and (vi) Green PMT to 100%. The TIFF images files and the quantification of fluorescence signal were obtained using Agilent Feature Extraction software version 11.0.1.1 to extract raw data and obtain QC reports.

### Transcriptomics data analysis

Data of the samples that pass quality control parameters were after subjecting to percentile normalization using GeneSpring GX 13.0 software (Agilent Technologies, UK). Genes were considered as differentially expressed with *p* values < 0.001 and fold change values of > |1.5|. Statistical analysis was performed using Benjamini-Hochberg False Discovery Rate. Statistically significant gene changes in each NP group were analyzed in terms of their associated molecular/cellular functions and representation in canonical pathways using Ingenuity Pathway Analysis software (IPA, v.39480507, release date September 2017, Qiagen Bioinformatics, Redwood City, USA).

### SP3 cell proteomics

Single-pot solid-phase-enhanced sample preparation (SP3), using commercially available carboxylate-modified magnetic beads, was employed to analyze the NR8383 cells global proteome (Hughes et al. 2014). Briefly, cells were exposed for 24 h to ¼ IC_50_ of ZnO and ZnFe_2_O_4_ NPs. Thereafter, cell disruption was performed in 6 M urea, 2 M thiourea, and 50 mM MOPS containing lysis buffer. Samples were then reduced and alkylated by DTT and iodoacetamide (IAA) respectively. Both hydrophobic and hydrophilic Sera-Mag SpeedBead carboxylate-modified magnetic particles (GE Healthcare, cat nos. 65152105050250 and 45152105050250) were mixed in a 1:1 ratio and were added to each sample. Once immobilized on the hydrophobic and hydrophilic carboxylate-modifed magnetic beads, proteins and peptides were rinsed with a combination of ethanol and acetonitrile mixture to efficiently remove contaminating agents. After rinsing, proteins and peptides are eluted from the magnetic beads by adding a mass spectrometry (MS) solution (Fisher Scientific, cat no. 12321D).

### Mass spectrometry and proteomic analysis

Each sample out the three biological replicates was run in triplicate on a Thermo Scientific Q Exactive mass spectrometer connected to a Dionex Ultimate 3000 (RSLCnano) chromatography system. Each sample was loaded onto a fused silica emitter (75 μm ID), using a laser puller (Sutter Instruments P2000, Novato, CA, USA), packed with Reprocil Pur (Dr Maisch, Ammerbuch-Entringen, Germany). Peptides were trapped on C18 columns (1.9 μm; 12 cm in length). Tryptic peptide elution was performed with a gradient of mobile phase media with 0.1 of formic acid and was separated by an increasing acetonitrile gradient over 60 min at a flow rate of 250 nL/min direct into a Q-Exactive MS. The MS was operated in positive ion mode with a capillary temperature of 320 °C, and with a potential of 2300 V applied to the frit. All data were acquired while operating in automatic data-dependent switching mode. A high-resolution (70,000) MS scan (300–1600 m/z) was performed using the Q Exactive to select the 12 most intense ions prior to MS/MS analysis using high-energy collision dissociation (HCD). Data acquisition for protein identification and quantification was done by MaxLFQ (Cox et al. 2014) searching with the MaxQuant version 1.5 reference proteome database (Uniprot). Modifications included C carbamylation (fixed) and M oxidation (variable). We used three biological replicate experiments per condition to ensure the quality of the quantification results. Two ratios of the intensities of the peptides with adjusted *p* value less than 0.05 were used to determine the expression ratio for each protein between exposed and control samples. Ratios over 1.5 and less than 1 were selected.

## Results

### Zinc, zinc iron, and iron oxide nanoparticle properties

**Fig. 1:**
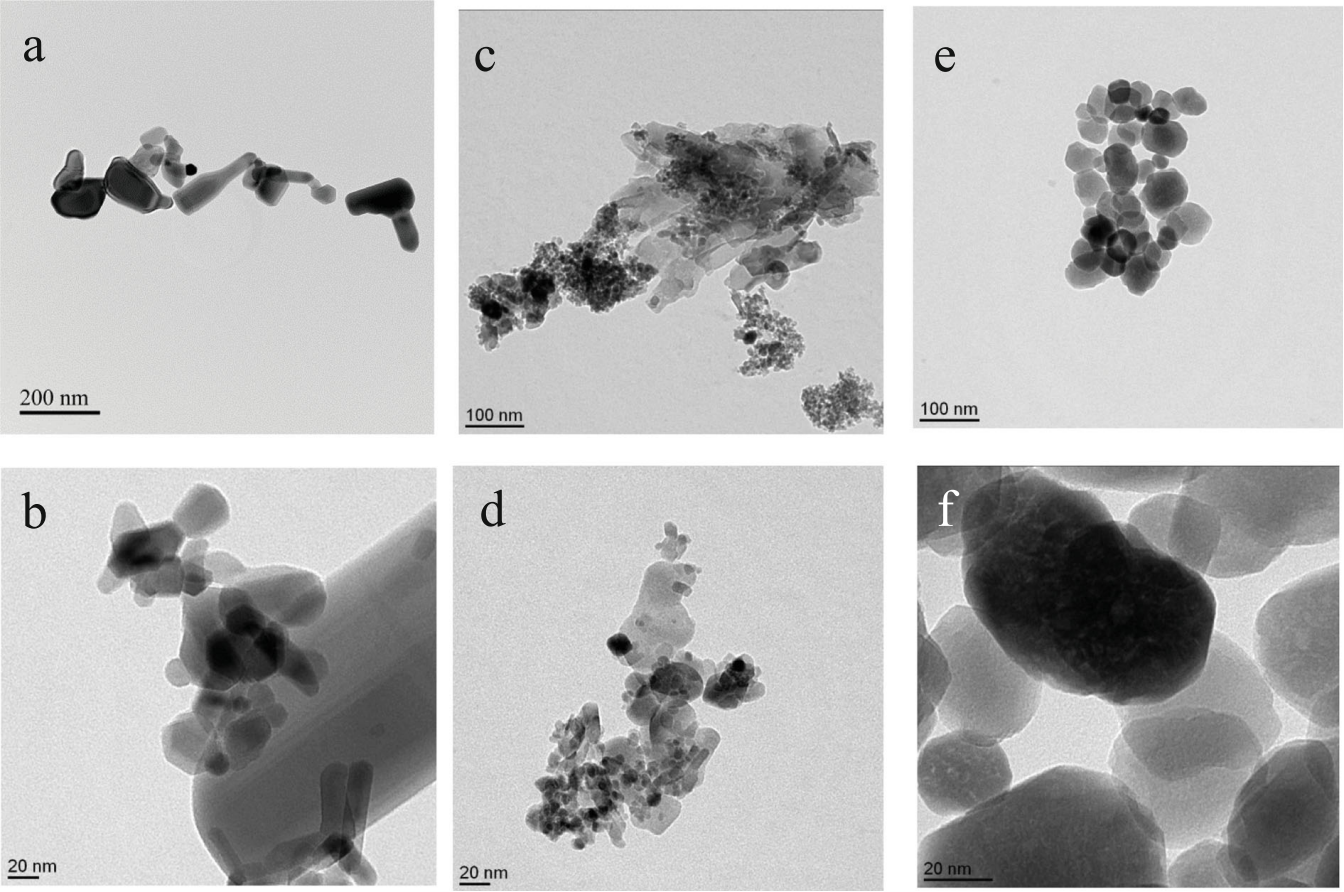
Transmission electron microscopy images of zinc oxide NPs (**a**, **b**), zinc iron oxide (**c**, **d**), and iron oxide (**e**, **f**)

To explore the effects of zinc and iron particles in vitro, the morphology of ZnO, ZnFe_2_O_4_, and Fe_2_O_3_ NPs was assessed by TEM. The sample of ZnO particles (Fig. 1a, b) exhibited as expected a diversity of forms such as rectangular, rod, spherical, and irregular shapes, whereas ZnFe_2_O_4_ NPs displayed a combination of the spherical shape specific to the Fe_2_O_3_ NPs and nanosheets specific to ZnO NPs (Fig. 1c, d). Fe_2_O_3_ NPs revealed a spherical shape (Fig. 1e, f). Characterization of NPs in DMEM FBS-free was measured by DLS that is widely used to determine the size of Brownian NPs in colloidal suspension (Lynch and Dawson 2008). Indeed, measured secondary sizes represented by the hydrodynamic diameters (*D*_H_) (Table 2) were as follows: 296 ± 4, 283 ± 36, and 357 ± 8 nm for the ZnO NPs at respectively concentrations of 6.25, 50, and 200 μg/mL. At the same increasing concentrations, (i) ZnFe_2_O_4_*D*_H_ were 71 ± 18, 224 ± 14, and 338 ± 23 nm; (ii) Fe_2_O_3_*D*_H_ were 133 ± 3, 144 ± 2, and 137 ± 1 nm. Furthermore, at the same concentrations, zeta-potential were (i) for ZnO − 19 ± 1, − 13, and − 31 mV; (ii) for ZnFe_2_O_4_ − 17, − 23, and −21 mV; (iii) for Fe_2_O_3_ 27 ± 1, 6, and 36 mV. The results of *D*_H_ characterization by DLS, at the lowest concentration (6.25 μg/mL), were the closest to the primary size characterization by TEM for all NPs. Indeed, by increasing concentration, it is likely that agglomerates are formed. It is important to mention that the specific surface area of ZnFe_2_O_4_ was 3.1 times higher than the one of Fe_2_O_3_ NPs and 7.3 times higher than the one of ZnO NPs (Table 1).

**Table 2:** Hydrodynamic diameter (*D*_H_) and zeta-potential (ζ) of ZnO, ZnFe_2_O_3_, and Fe_2_O_3_ by dynamic light scattering (DLS)

	ZnO	ZnFe_2_O_4_	Fe_2_O_3_
6.25 μg/mL NPs in cell medium			
*D*_H_ (n m)	296 ± 4	71 ± 18	133 ± 3
*ζ* (mV)	−19 ± 1	−17	27 ± 1
50 μg/mL NPs in cell medium			
*D*_H_ (nm)	283 ± 36	224 ± 14	144 ± 2
*ζ* (mV)	−13	−23	6
200 μg/mL NPs in cell medium			
*D*_H_ (n m)	357 ± 8	338 ± 23	137 ± 1
*ζ* (mV)	−31	−21	36

### ZnO and ZnFe_2_O_4_ nanoparticles reduced the viability of NR8383 cells

**Fig. 2:**
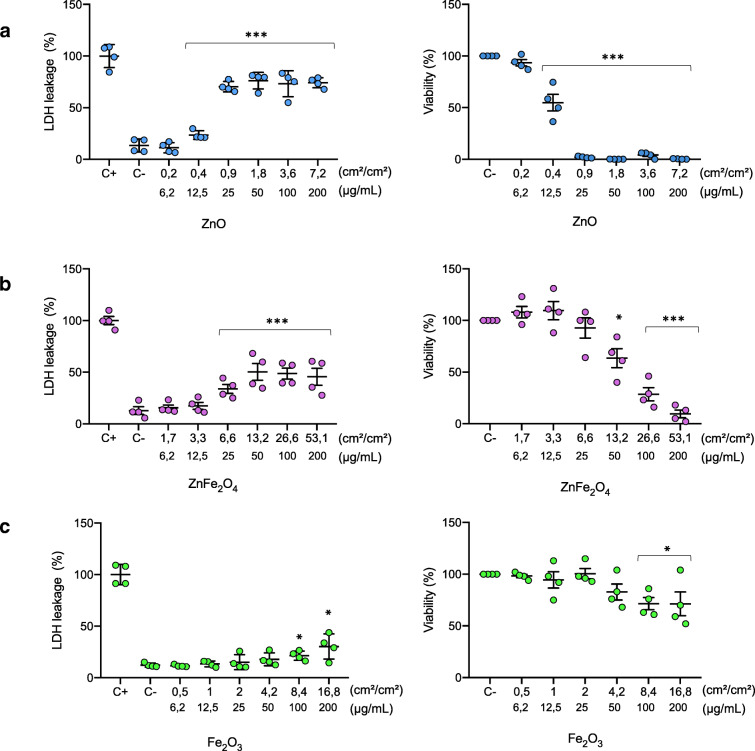
Cytotoxicity of ZnO NPs (**a**), ZnFe_2_O_4_ (**b**), and Fe_2_O_3_ (**c**). Cytotoxicity was determined after 24 h exposure of NR8383 to the panel of studied nanoparticles. At the left, the induction of membrane damage after cell exposure to different studied nanoparticles by measuring the level of extracellular LDH. At the right, action of ZnO, ZnFe_2_O_4_, and Fe_2_O_3_ on NR8383 metabolic activity measured by WST1 assay. Calculated IC_50_, based on WST-1 assay data, for ZnO, ZnFe_2_O_4_, and Fe_2_O_3_ were respectively 16, 68, and more than 200 μg/mL

The potential cytotoxicity of the three NPs on NR8383 cells after 24 h of exposure was determined by measurements of LDH leakage and WST-1 assay. When the plasma membrane is impaired, LDH diffuses into extracellular media, and extracellular LDH increases with the extent of cytotoxicity of the NP. In each experiment, the positive control was triton-exposed NR8383 cells. A dose-dependent LDH leakage was observed for NR8383 cells exposed to ZnO and ZnFe_2_O_4_ NP up to 50 μg/mL. Cells exposed to 50 μg/mL of ZnO and ZnFe_2_O_4_ displayed respectively 75 and 50% increases of extracellular LDH, a dose reaching a plateau. One should notice that only the highest dose of Fe_2_O_3_ induced a 30% release of LDH (Fig. 2a). In conclusion, among the three NPs, ZnO exhibited the highest cytotoxic effect on NR8383 cell membrane, thus conferring for ZnO the highest toxic potential despite the lowest specific surface area.

WST-1 assay measures the metabolic activity by giving different absorption spectra of formazan formed by reduction of tetrazolium by mitochondrial dehydrogenase. Our results showed that ZnO NPs induced a strong decrease in cell viability as shown in Fig. 2b. As a matter of fact, at a concentration of 12.5 μg/mL of ZnO NPs, only 54% of cells remained metabolically active, while a complete cytotoxicity was observed up to 25 μg/mL. A decrease in the viability of cells exposed to ZnFe_2_O_4_ NPs was also observed. Indeed, after exposure of NR8383 to 50 and 200 μg/mL of ZnFe_2_O_4_ NPs, there remained 64% and 10% of metabolic active cells, respectively. NR8383 cell viability was not affected by Fe_2_O_3_ NPs up to a concentration of 25 μg/mL but decreased continuously in a dose-dependent manner (Fig. 2b). However, Fe_2_O_3_ NPs seemed to be less cytotoxic in our model compared with the two other NPs. Indeed, NR8383 cell viability decreased only to 30% in the presence of the highest Fe_2_O_3_ NPs (200 μg/mL) used in our study (Fig. 2b). Both in LDH and WST-1 assays were results concordant, demonstrating that the presence of Zn in NPs is the main factor of main toxicity (Fig. 2a).

Based on the WST-1 assay data, the calculated IC_50_ was 16, 68, and > 200 μg/mL for respectively ZnO, ZnFe_2_O_4_, and Fe_2_O_3_ NPs, allowing us to set up the optimal sub-toxic concentrations for each NP for the following experiments.

### Effective dosimetry for nano-bio interactions

Based on the dose-response toxicity results for NR8383 cells exposed to ZnO, ZnFe_2_O_4_, and Fe_2_O_3_, we deliberately decided to deepen our study with NP whose IC_50_ were measurable, namely ZnO and ZnFe_2_O_4_ NPs. Therefore, we evaluated the amount of cytotoxic NPs in contact with the cells by measuring the zinc (Zn) and iron (Fe) contents in the cell layers by ICP-OES. This measurement was carried out on NR8383 cells exposed for 24 h to both ¼ IC_50_ (4 and 17 μg/mL) and IC_50_ (16 and 68 μg/mL) of ZnO and ZnFe_2_O_4_, respectively. Table 3 indicates that Zn concentrations in/onto NR8383-cell were respectively 62.1 ± 1.5 and 130.7 ± 0.4 ng/mL, for ¼ IC_50_ and IC_50_ of ZnO. For ZnFe_2_O_4_ NPs, Zn concentrations in/onto cells were 16.1 ± 0.3 and 18.2 ± 0.1 ng/mL, for ¼ IC_50_ and IC_50_ of ZnFe_2_O_4_, respectively. Also, Fe concentrations were 82.7 ± 0.2 and 78.2 ± 0.3 ng/mL for ¼ IC_50_ and IC_50_ of ZnFe_2_O_4_, respectively. The results showed that free Zn in contact with cells was more abundant following exposure to ZnO NPs than the exposure to ZnFe_2_O_4_ NPs, conferring the primary role of free Zn in cell viability.

**Table 3:** Measure of zinc (Zn) and iron (Fe) in/onto NR8383 cells by inductively coupled plasma-optical emission spectrometer

	ZnO		ZnFe_2_O_4_	
	¼ IC_50_	IC_50_	¼ IC_50_	IC_50_
Theoretical NP concentration (μg/mL)	4	16	17	68
Measured Zn concentration (ng/mL) in cell phase	62.1 ± 1.5	130.7 ± 0.4	16.1 ± 0.3	18.2v0.1
Measured Fe concentration (ng/mL) in cell phase	n.d	n.d	82.7 ± 0.2	78.2 ± 0.3

### Action on transcriptome

**Fig. 3:**
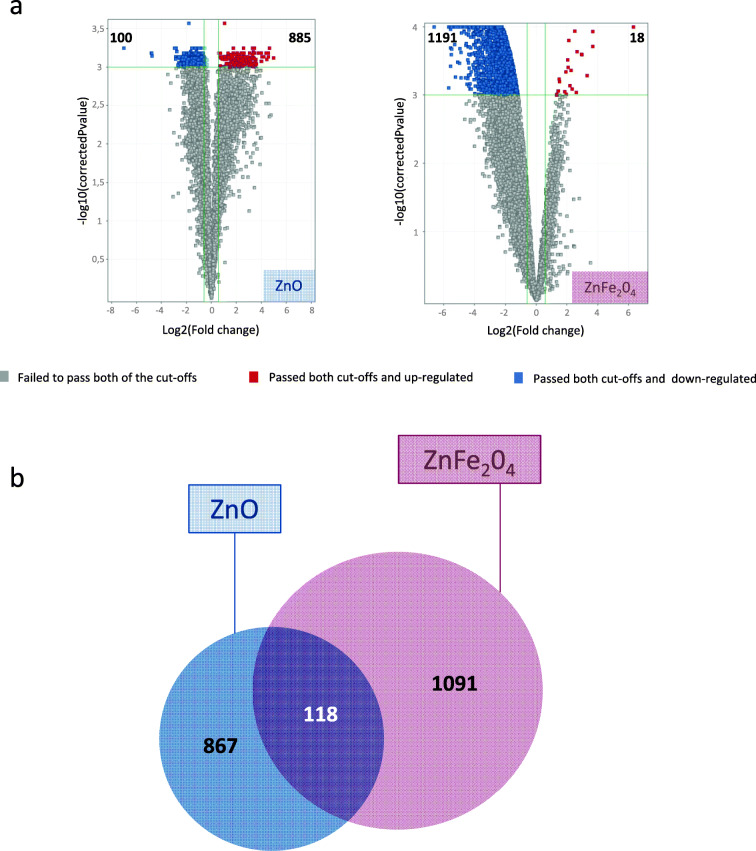
Differentially expressed genes (DEG) of NR8383 cells exposed to ¼ IC_50_ of ZnO and ZnFe_2_O_3_ NPs during 4 h. Two cutoffs were applied: statistical test Benjamini-Hochberg corrected at *p* value < 0.001 and fold changes > |1,5|. **a** Representative volcano plots of differentially overexpressed (in red) and downexpressed (in blue) genes, for each exposure condition. Numbers of DEG are indicated. **b** Venn diagram showing common differential gene expression between cells exposed to ZnO and ZnFe_2_O_3_ NPs

To identify key pathways linked to the response of NR8383 to sub-toxic doses of NP exposure, transcriptomics experiments were conducted by their exposure to ZnO and ZnFe_2_O_4_ at ¼ IC_50_ dose for 4 h. Results showed 985 and 1209 DEG that were revealed after the exposure to ZnO and ZnFe_2_O_4_ NPs, respectively (Fig. 3a).

**Fig. 4:**
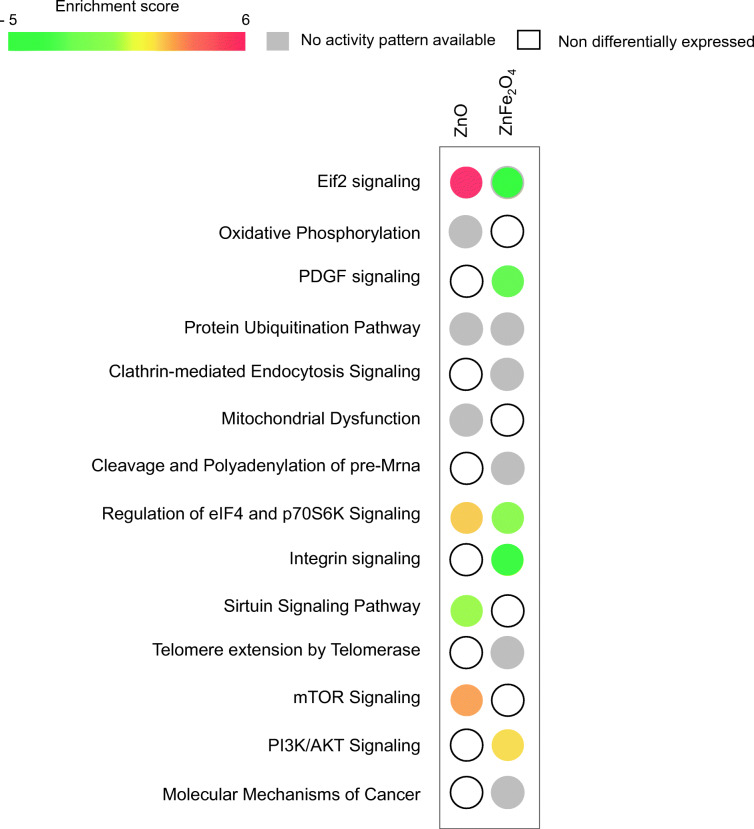
Heatmap showing 14 deregulated canonical pathways after ZnO and ZnFe_2_O_4_ NP exposures. Statistical test Benjamini-Hochberg corrected at *p* value < 0.001 and fold changes = |1,5|. IPA software based on *p* < 0.05. Deregulated pathway more significant from left to right

Following exposure to ZnO, the proportion of downregulated genes was 100 and upregulated genes was 885. Interestingly, exposure to ZnFe_2_O_4_ led to a higher fraction of downregulated genes, e.g., 1191 versus only 18 upregulated genes (Fig. 3a). Our results highlighted 118 common DEG to both exposure conditions, whereas 867 DEG were specific to ZnO and 1091 DEG were specific to ZnFe_2_O_4_ (Fig. 3b). To determine the main affected pathways, all DEG were further analyzed using IPA® software. The heatmap (Fig. 4) showed 14 deregulated canonical pathways of which were activated. Indeed, eIF2 and both eIF4/p70S6K and mTOR signaling pathways were predicted to be activated in cells exposed to ZnO but not following exposure to ZnFe_2_O_4_. On the contrary, eIF2, both eIF4/p70S6K, PDGF, and integrin signaling were predicted to be inhibited in cells exposed to ZnFe_2_O_4_.

Finally, the most significant deregulated common pathways in ¼ IC_50_ exposure to ZnO and ZnFe_2_O_4_ NP were those involved in the homeostasis of cellular protein production/degradation. What differs is the predictive trend of activation or inhibition of these pathways calculated by IPA.

**Fig. 5:**
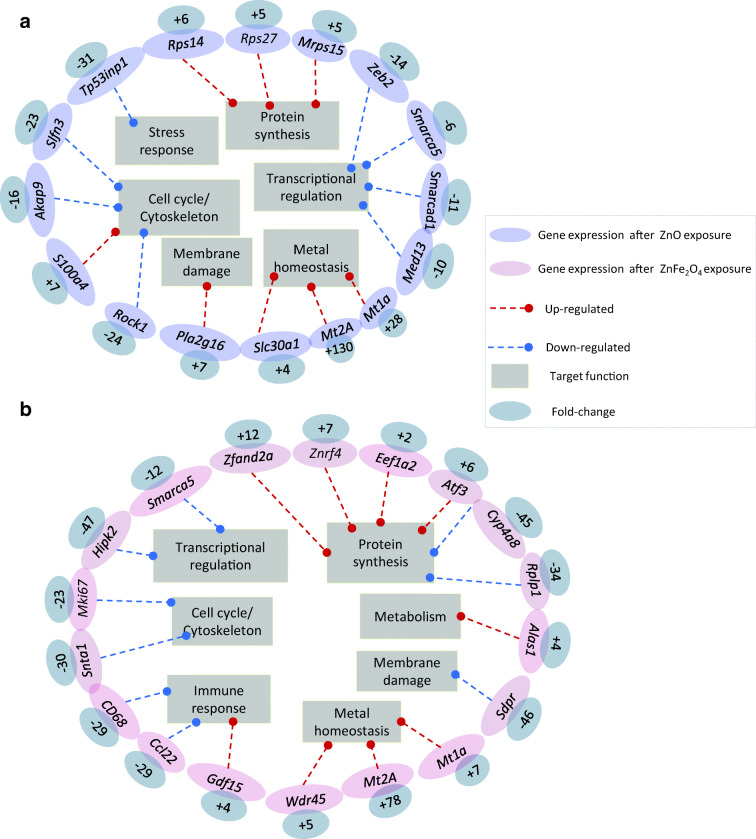
The main strongly deregulated genes in NR8383 cells exposed to ¼ IC_50_ of ZnO NPs (**a**) and ¼ IC_50_ of ZnFe_2_O_4_ NPs (**b**) during 4 h. Statistical test was Benjamini-Hochberg corrected at *p* value < 0.001. Both blue (**a**) and pink (**b**) colors are representing the ZnO and ZnFe_2_O_4_ NP deregulated genes. The fold change values are in the gray circles linked to the corresponding genes. The red and blue arrows indicate overexpression and underexpression of linked genes, respectively. Finally, the functions involved are arranged in rectangles

To go further and to delineate a potential gene signature, we focused on the most up- and most downregulated genes in NR8383 exposed to ZnO NPs (Fig. 5a) and ZnFe_2_O_4_ NPs (Fig. 5b). Not surprisingly, *Mt1a* and *Mt2A*, both metallothioneins involved in metal homeostasis, were among the most upregulated genes in both conditions (FC respectively + 28 and + 130 for ZnO; FC respectively + 7 and + 78, for ZnFe_2_O_4_). *Slc30a1* (FC + 4) and *Wdr45* (FC + 5) also involved in metal homeostasis were also highly upregulated, but only after exposure to ZnO or to ZnFe_2_O_4_, respectively. ZnO specific gene signature further englobed the overexpression of *Pla2g16* (FC + 7) a membrane damage sensor; *S100a4* (FC + 7) involved in macrophage-induced lung fibrosis (Li et al. 2018; Zhang et al. 2018); *Rps14* (FC + 6), *Rps27* (FC + 5), and *Mrps15* (FC + 5), three protein synthesis regulators. Besides, ZnO exposure signature was also characterized by a downregulation of *Tp53inp* (FC − 31); a stress response mediator, *Slfn3* (FC − 23), *Akap9* (FC − 16), and *Rock1* (FC − 24), three cell cycle/cytoskeleton regulators; and *Zeb2* (FC − 14), *Smarca5* (FC − 6), *Smarcad1* (FC − 11), and *Med13* (FC − 10), four transcriptional regulators. NR8383 cell specific gene signature following exposure to a sub-lethal dose of ZnFe_2_O_4_ was marked by the deregulation of genes involved in protein synthesis such as *Eef1a2* (FC + 2), *Atf3* (FC + 6), *Cyp4a8* (FC − 45), and *Rpl1* (FC − 34), as well as both zinc-finger proteins *Zfand2a* (FC + 12) and *Znrf4* (FC + 7), in membrane damage sensing, such as *Sdpr* (FC − 46), and in cell cycle/cytoskeleton regulation such as *Mki67* (FC − 23) and *Snta1* (FC − 30). Genes encoded for transcriptional regulators, such as *Smarca5* (FC − 12) and *Hipk2* (FC − 47), and immune response effectors, such as *CD68* (FC − 29), *Ccl22* (FC − 29), and *Gdf15* (FC + 4), were also deregulated. The exposure of NR8383 to ZnFe_2_O_4_ NPs further showed an overexpression of *Alas1* (FC +4) involved in heme biosynthesis (Fig. 5).

### Effect on proteome

**Fig. 6:**
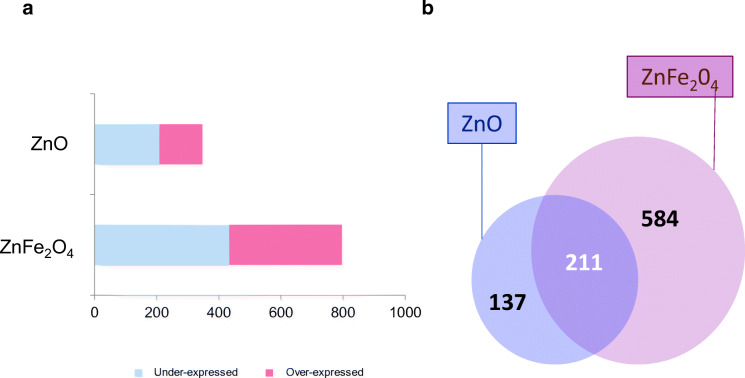
Histogram of proteomic data (**a**). Data for the total differentially produced proteins (DPP). Venn diagram (**b**) showing common DPP between cells exposed to ZnO and ZnFe_2_O_3_ NPs

Differential protein expression from NR8383 cells exposed for 24 h to ¼ IC_50_ of ZnO and ZnFe_2_O_4_ NPs was further investigated by using mass spectrometry-based proteomics approach. Mass spectrometry protein identification and quantification indicated 348 differentially expressed proteins (DEP) in exposed cells to ZnO compared with unexposed ones with *p* value < 0.05 and 1.5 < ratio < 1 (Fig. 6). Cells exposed to ¼ IC_50_ ZnFe_2_O_4_ NPs showed total 795 DEP (Fig. 6). Furthermore, 211 DEP were common to both exposure conditions. NR8383 exposed to ZnO NPs showed 137 specific DEP while ZnFe_2_O_4_ NP exposure led to 584 specific DEP (Fig. 6b). Both gene and protein expressions were more abundant after ZnFe_2_O_4_ exposure and displayed more underexpressed elements than overexpressed ones (Figs. 3a and 6a).

To assess the molecular pathways, we also analyzed DEP by using the IPA software. Table 4 indicates the final ranking of the selected canonical pathways (*p* value ≤ 0.05) together with the number of deregulated proteins involved for each condition, and the enrichment score for each significantly deregulated pathway. Indeed, protein synthesis with the eIF2 signaling pathway, stress response with mitochondrial dysfunction, and sirtuin signaling were the most affected functions. Indeed, 16 and 30 DEP were found to be linked to eIF2 signaling following exposure to NPs ZnO and ZnFe_2_O_4_, respectively.

**Table 4:**
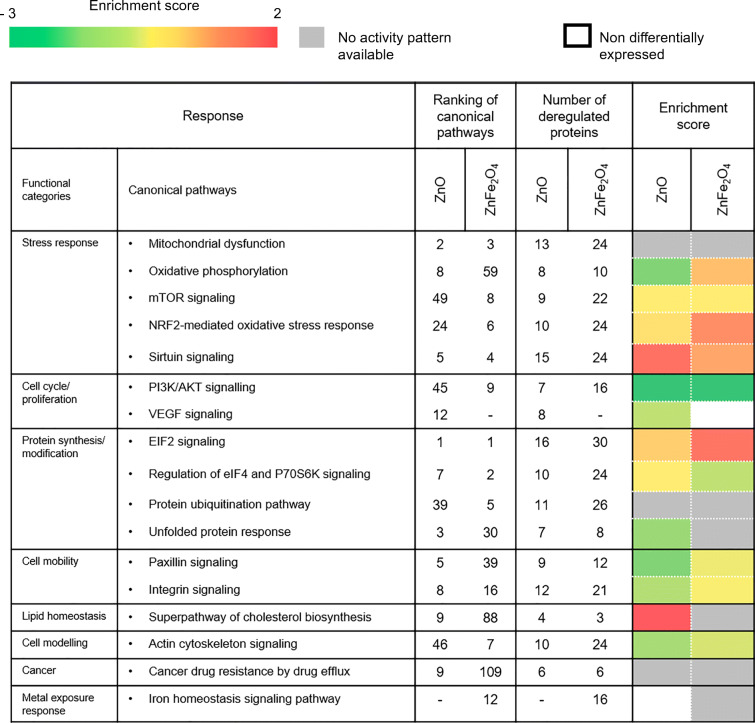
Ranking of selected canonical pathways and number of deregulated produced proteins involved in each condition

Interestingly, IPA analysis revealed a trend toward activation of the eIF2 pathway following both exposures, in the protein synthesis cluster. Similarly, in the stress response cluster, 15 and 24 of DEP were linked to sirtuin signaling and 13 and 24 of DEP were ranked in mitochondrial dysfunction in cells exposed to ZnO and ZnFe_2_O_4_ NPs, respectively.

The superpathway of cholesterol biosynthesis in lipid biosynthesis cluster revealed a positive enrichment score with following exposure to ZnO NPs namely a predicted activation, as sirtuin pathway, as well as deregulation of VEGF signaling that was specific to ZnO exposure. On the other hand, this pathway was not suggested as activated following exposure to ZnFe_2_O_4_ NPs. In addition, the metal exposure response included the iron homeostasis signaling pathway which was deregulated only after exposure to ZnFe_2_O_4_ NPs, not after exposure to ZnO NPs, confirming a role of iron at an ionic level.

**Fig. 7:**
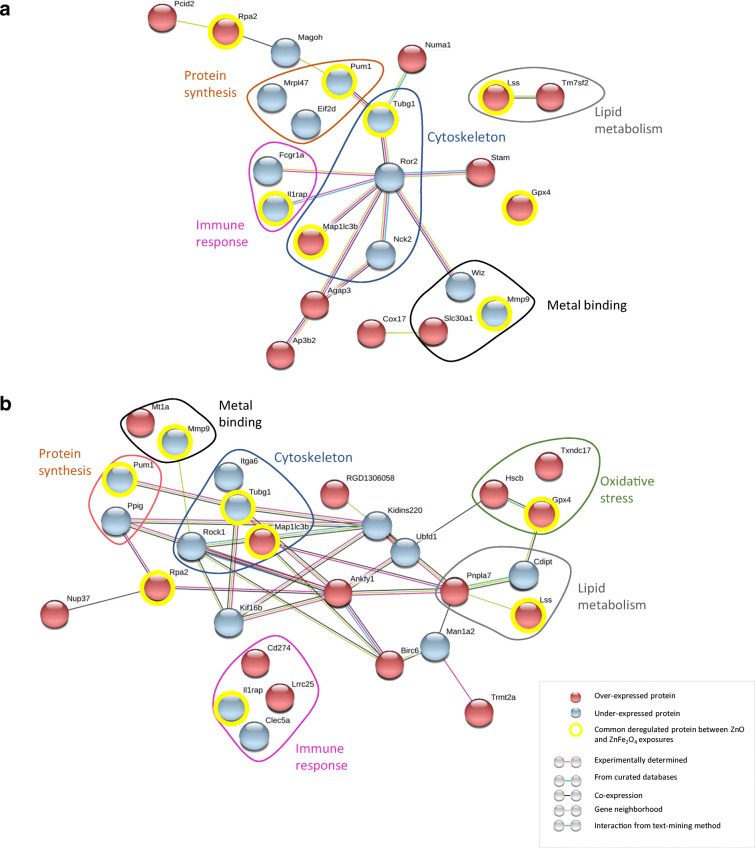
The main strongly deregulated proteins in NR8383 cells exposed to both ¼ IC_50_ doses of ZnO NPs (**a**) and ZnFe_2_O_4_ NPs (**b**) during 24 h. Proteins ratio (**a**): 16 × 10^6^ ≤ R_ZnO_ ≤ 0.18; protein ratio (**b**) 6 × 10^6^ ≤ R_ZnFe2O4_ ≤ 2.6 × 10^−7^

Moreover, the most deregulated proteins, namely 23 after ZnO exposure and 28 after ZnFe_2_O_4_ exposure, have been analyzed on STRING 10.5 version (http://string-db.org/). Overexpressed (in red) and underexpressed (in blue) proteins revealed six common clusters following ZnO (Fig. 7a) and ZnFe_2_O_4_ exposures (Fig. 7b). Those clusters were referred to as “cytoskeleton modeling” with 2 proteins that were identically deregulated after both exposures, “oxidative stress,” “protein synthesis” with mainly underexpressed proteins, “immune response,” and “zinc-related proteins.”

From the list of these deregulated proteins, eight proteins were common following both exposures studied, namely Il1rap, Gpx4, Lss, Mmp9, Pum1, Rpa2, Tubg1, and Map1lc3b (circled in yellow in Fig. 7). Also, comparison of both DEP (Fig. 7) and DEG (Fig. 5) showed that Slc30a1 and Mt1a were overexpressed at transcriptional and protein levels in cells exposed to ZnO and ZnFe_2_O_4_ NPs, respectively.

## Discussion

The cellular response to exposure to sub-toxic doses of nanoparticles of metal oxides has been investigated using a rat alveolar pulmonary NR8383 macrophage model. The aim was to decipher transcriptional and proteomic signatures in cells.

Cell viability analyses following exposure of NR8383 macrophages to increasing doses of ZnO, ZnFe_2_O_4_, and Fe_2_O_3_ clearly highlight the higher cytotoxic potential of ZnO, followed by ZnFe_2_O_4_ and thereafter Fe_2_O_3._

Physicochemical properties of NPs, especially size and specific surface area, have been reported to affect cell viability. Indeed, nanoscaled particles are known to be significantly more toxic than microscaled particles (Li et al. 2018; Zhang et al. 2018). Here, in our experimental conditions, we showed that ZnO NPs display the larger primary size and the lower specific area, suggesting that ZnO toxicity in this model could be attributed to other properties such as their solubility, morphology, or presence of Zn. Our data are in agreement with the reported toxicity of ZnO in rat epithelial and pulmonary cell models as well as in in PMA-differentiated THP-1 monocytic cells to a macrophage-like phenotype (Safar et al. 2018; Wiemann et al. 2016). Moreover, the ZnO NPs toxicity potential seems to be cell-type dependent as similar concentrations of ZnO NPs do not lead to any cytotoxicity in exposed human monocyte THP1 cells and in human umbilical vein endothelial cells (Chen et al. 2017; Gong et al. 2017). However, this cell-type–dependent toxicity of ZnO needs to be confirmed on a larger panel of cells and tissues. This would be a unique opportunity to better define the mechanism of adverse health effects on exposure to ZnO. In our model, Fe_2_O_3_ NPs affect NR8383 macrophages’ viability only at the highest dose used (200 μg/mL), supporting the anterior finding that Fe_2_O_3_ is rather a “passive” NPs (Wiemann et al. 2016). Besides, ZnFe_2_O_4_ NPs demonstrate an intermediate dose-dependent effect on NR8383 cells, between those of ZnO and Fe_2_O_3_ NPs. As ZnFe_2_O_4_ NPs are composed of 5–10 wt% of ZnO and 10–15 wt% Fe_2_O_3_, we could postulate that ZnFe_2_O_4_ toxicity relies mainly on the toxic potential of ZnO. As (i) WST-1 may be reduced by NADPH oxidases and (ii) ZnO may activate superoxide formation by triggering p47phox, we assayed cell viability using resazurin reduction (Alamar blue™), that is reduced by the first enzyme of the mitochondrial respiratory chain, NADPH dehydrogenase. Fifty percent of the latter enzyme which occurred with 5.5 μg/mL of ZnO correspond to 16.0 μg/mL for succinate dehydrogenase (data not shown). One can reasonably assume that the IC_50_ of cell viability is comprised between both values. Furthermore, when IC_50_ of viability is taken into account, the average of IC_50%_ was 10.8 ± 5.23 μg/mL. For ZnFe_2_O_4_, IC_50_ were 98.0, 58, and 68 when respectively calculated with Spearman and Karber method of resofurin, LDH, and tetrazolium salts. The calculated average was 74.7 ± 20.9 μg/mL which was statistically different at *p* < 0.05 of the average of ZnO, demonstrating higher in vitro toxicity of the latter NPs.

Following exposure to sub-toxic concentrations of NPs (¼ IC_50_), molecular pathways are supposed to produce an adaptive response in order to maintain the cellular homeostasis in front of the induced disturbances and may confer a resistance or an adaptation following the aggression of the toxic (Jennings 2013). Therefore, the pathways of both toxicity and adaptive responses are important to explore, in order to understand the exact cellular reaction to NPs exposure. So, we realized a transcriptome and a proteome exploration after 4 h and 24 h of exposure of NR8383 cells to the ¼IC_50_ of ZnO and ZnFe_2_O_4_ NPs, respectively. As far as we know, this is the first transcriptome and proteome study for ZnFe_2_O_4_ NPs reported.

One of the aims of the work was to decipher key molecular pathways and/or functions associated with NR8383 cell response to sub-toxic doses of ZnO and ZnFe_2_O_4_ NPs. Therefore, the most deregulated genes were involved in metal homeostasis. Indeed, the metallothioneins *Mt1a* and *Mt2a* were commonly overexpressed at the transcriptional level. Exposure to ZnO and ZnFe_2_O_4_ NPs leads also to specific deregulation of other effectors involved in metal homeostasis. Indeed, *Slc30a1* and *Wdr45* were respectively increased following exposure to ZnO and ZnFe_2_O_4_ NPs. Noteworthy is the upregulation of Zn transporter *Slc30a1*after exposure to Zn element which has already been observed following an in vivo exposure of Zn to piglets (Chai et al. 2014). Moreover, the higher amount of Zn element found in cells exposed to ZnO NPs, in addition to their soluble potential, could be related to the transient expression of Zn-dependent proteins, such as those encoded by *Slc30a* genes, *Zfand2b*, *Zgpat*, *Zfp637*, or *Zfyve19* (Table S1a and S1b).

A metal exposure response was also evidenced following the analysis of proteomic data with IPA™ software; *Mmp9* was highly downregulated for both studied exposures (Fig. 7). The iron homeostasis signaling pathway was only deregulated after exposure to ZnFe_2_O_4_ (Table 4). Our results are in agreement with those of Tuomela et al. (2013) who demonstrated an overexpression of different metallothioneins as a gene signature of three different immune cell lines after the exposure to ZnO NPs. Other recent studies revealed as well the expression of MTs after exposure to Zn, nano-ZnO, or micro-ZnO (Chai et al. 2014; Safar et al. 2018; Yang et al. 2017).

The overexpression of metallothioneins is an indicator of an adaptive response that plays a role in defense mechanisms against oxidative stress. Indeed, they are known to efficiently trap oxygen species (ROS) as well as Zn ions (Vallee 1995; Sato and Kondoh 2002; Vašák 2005; Lindeque et al. 2010). In this study, metallothioneins were not the only indicator of oxidative stress. After 24 h of exposure to NPs ZnO and ZnFe_2_O_4_, deregulated proteins were mainly involved in mitochondrial dysfunction, oxidative phosphorylation, and sirtuin pathways. Our data are in agreement with previous studies (Aude-Garcia et al. 2016; Chevallet et al. 2016; George and Ahmad 2016; Niska et al. 2015). Altogether, these findings suggest that the regulation of these pathways might help cells capture and fix Zn^2+^ and reduce the oxidative effect induced by ZnO and ZnFe_2_O_4_ NPs.

One of the consequences of oxidative stress is the accumulation of cholesterol (Gesquière et al. 1999). Indeed, overexpression of proteins involved in cholesterol synthesis has been revealed in both studied exposures. At sub-toxic doses of exposure to ZnO and ZnFe_2_O_4_ NPs for 24 h, the overexpression of the Gpx4 protein could be a response to cholesterol synthesis. The Gpx4 enzyme has important biological functions; it is particularly known for its action against lipid peroxidation of cells and its inhibition induces cell death by ferroptosis (Maiorino et al. 2018). All these results suggest that the induction of oxidative stress would cause lipid accumulation and drop antiperoxidative capacity of the cells, which could justify the overexpression of the Gpx4 enzyme.

Moreover, cholesterol production control occurs in the endoplasmic reticulum (ER). Thus, when the mechanism of cholesterol production is altered, it induces ER stress (Sozen and Ozer 2017).

In this present study, we revealed the significant deregulation of different pathways related to protein homeostasis, namely eIF2, eIf4/p70S6K, and unfolded protein response signaling. Anterior proteomic studies showed that ZnO NPs induce disturbance of proteins involved in protein synthesis by deregulating structural constituent of ribosomes in human monocyte-derived macrophage but not in Jurkat cells (Tuomela et al. 2013). The fundamental difference between these findings concerns the cell line type used. In our study, the main altered gene expression was involved in the ribosome biogenesis and several translation initiation factors such as *eIF3k* and *eIF2β*, as well as 40S and 60S ribosomal subunits such as *Rpl36a*, *Rpl9*, or *Rpl14* genes, and transcription factors such as *Atf7* and *Atf3*. It should be noticed that the latest one, *Atf3*, has a binding site on the promoter sequence of the two upregulated genes that code for zinc-finger proteins *Zfand2A* and *Znrf4* after ZnFe_2_O_4_ exposure. These zinc-finger proteins are also involved in the protein synthesis cluster (LifeMap sciences, GeneCards Suite® Knowledgebase, version 4.9).

It is also important to note that the proteomics results revealed a specific and significant deregulation of the VEGF pathway following exposure of cells to ZnO NPs through inhibition of Acta1 and Actn1, among others. This pathway was predicted to be inhibited in NR8383 cells following exposure to ZnO NPs during 24 h (Table S2). Coherently, Tada-Oikawa et al. (2015) described the vascular endothelial growth factor pathway to be negatively regulated by ZnO NPs on human endothelial cells suggesting that these effects are based on the concentration of released Zn^2+^. In contrast, other studies have demonstrated activation of the VEGF pathway following exposure of human dermal fibroblasts to ZnO NPs (Augustine et al. 2014). These differences could be due to the nature of exposed cell lines that are from two different embryonic origins. Therefore, inhibition of the VEGF pathway in NR8383 cells suggests a cytoskeleton defect and may be a migration default induced by ZnO NPs. As far as we know, no previous study has shown a disturbance of the VEGF pathway by ZnO NPs in macrophages.

## Conclusion

Altogether, our results along with previous reports clearly demonstrate the hazard effects associated to the exposure of ZnO and ZnFe_2_O_4_ NPs. These compounds are already used in several products, from toothpaste to antibacterial gels and food, without a deep knowledge of how the human body could respond to exposure, whether short or long term. From the results obtained, ZnO NPs are by far the most cytotoxic NPs of the three NPs studied on NR8383, followed by ZnFe_2_O_4_ NPs and then Fe_2_O_3_ NPs. Cytotoxicity is related to the presence of the Zn element.

Also, based on this study, we suggest the metallothioneins *Mt1a* and *Mt2A* as exposure biomarkers of both NPs ZnO and ZnFe_2_O_4_, biomarkers that were validated by Figueira et al. (2012). Moreover, according to the transcriptome and proteome profiles, ZnO and ZnFe_2_O_4_ NPs induce ER stress that could be a molecular initiating event. This is highlighted by the deregulation of eIF2 pathway and dysfunction of cholesterol biosynthesis. Both NPs also induce oxidative stress by dysregulation of genes and proteins involved in mitochondrial functions, oxidative phosphorylation, and sirtuin homeostasis. However, the VEGF pathway was specific to ZnO exposure and iron homeostasis pathway specific to ZnFe_2_O_4_ exposure.

Finally, it is obvious that further studies under realistic biological conditions rather than artificial environment of culture cells should be done, to elucidate the mechanism of action of these nanometric structures.

## Electronic supplementary material

ESM 1 (DOCX 17.4 kb)
