# Supplementary material for: Protein and lipid homeostasis altered in rat macrophages after exposure to metallic oxide nanoparticles
Source: Cell Biol Toxicol. 2019 Jul 27;36(1):65–82. doi: 10.1007/s10565-019-09484-6 (PMC7051947; doi:10.1007/s10565-019-09484-6)
Supplement: Supplementary file 1 — (DOCX 17.4 kb) [file 10565_2019_9484_MOESM1_ESM.docx]

**Supplementary Data**

Supplementary Table 1a. Overexpressed genes that encode for zinc-dependent proteins in NR8383 cells after 4 h of exposure to 4 µg/mL of ZnO.

| **ID gene** | **Gene name** | **Fold Change** |
| --- | --- | --- |
| Slc30a | Solute carrier family 30 | 4,80 |
| Zfand2b | AN1-type zinc finger protein 2B | 4,20 |
| Zgpat | Zinc finger CCCH-type with G patch domain-containing protein | 3,39 |
| Zfp637 | Zinc fingerprotein 637 | 3,15 |
| Zfyve19 | Zinc Finger FYVE Domain-ContainingProtein 19 | 3,12 |
| Znrd1 | Zinc Ribbon Domain Containing 1 | 2,97 |
| Zbtb8os | Zinc Finger And BTB Domain Containing 8 Opposite Strand | 2,85 |
| Rbck1 | RANBP2-Type And C3HC4-Type Zinc Finger Containing 1 | 2,63 |
| Zfpl1 | Zinc Finger Protein Like 1 | 2,50 |
| Slc39a3 | Solute Carrier Family 39 Member 3 | 2,45 |
| Zfp524 | Zinc finger protein 524 | 2,45 |
| Slc39a4 | Solute Carrier Family 39 Member 4 | 2,42 |
| Zfp511 | Zinc finger protein 511 | 2,31 |
| Zdhhc16 | Zinc Finger DHHC-Type Containing 16 | 2,23 |
| Zdhhc12 | Zinc Finger DHHC-Type Containing 12 | 2,21 |
| Zfp956 | Zinc finger protein 956 | 2,12 |
| Zfp358 | Zinc finger protein 358 | 1,81 |
| Dzip1l | DAZ Interacting Zinc Finger Protein 1 Like | 1,59 |

Supplementary Table 1b. Overexpressed genes that encode for zinc-dependent proteins in NR8383 cells after 4 h of exposure to 17 µg/mL of ZnFe_2_O_4_.

| **ID gene** | **Gene name** | **Fold Change** |
| --- | --- | --- |
| Zfpl637 | Zinc FingerProteinLike623 | 3,57 |
| Znrf4 | Zinc And Ring Finger 4 | 7,69 |
| Zfand2a | Zinc Finger AN1-Type Containing 2A | 12,57 |

Supplementary Table 2. Profile of expressed proteins involved in VEGF pathway deregulated following exposure to 4 µg/mL of ZnO.

| **ID gene** | **Gene name** | **Ratio** |
| --- | --- | --- |
| RAP1B | Ras-related protein Rap-1b | 0,73 |
| RAF1 | RAF proto-oncogene serine/threonine-protein kinase | 1,98 |
| RALA | Ras-related protein Ral-A | 0,57 |
| YWHAE | Tyrosine 3-Monooxygenase/Tryptophan 5-Mono oxygenase Activation Protein Epsilon | 0,84 |
| RAP1A | Ras-related protein Rap-1A | 0,71 |
| ACTA1 | Actin, alpha skeletal muscle | 0,19 |
| ACTN1 | Alpha-actinin-1 | 0,92 |
| EIF2S2 | Eukaryotic translation initiation factor 2 subunit beta | 0,74 |
